# Aseptic Abscess of the Spleen as an Antecedent Manifestation of Behçet’s Disease

**DOI:** 10.7759/cureus.38375

**Published:** 2023-05-01

**Authors:** Mohamed Jazeer, Diroji Antony, Mayurathan Pakkiyaretnam

**Affiliations:** 1 Internal Medicine, Teaching Hospital Batticaloa, Batticaloa, LKA; 2 University Medical Unit, Teaching Hospital Batticaloa, Batticaloa, LKA; 3 Department of Clinical Sciences, Faculty of Health-Care Sciences, Eastern University of Sri Lanka, Batticaloa, LKA

**Keywords:** splenectomy, immunosuppression, splenic abscess, aseptic abscess, behçet’s disease

## Abstract

Behçet’s disease (BD) is a multisystem autoimmune vasculitis that manifests as oral and genital ulcers with varying degrees of dermatological and ocular involvement. Aseptic splenic abscesses are a rare entity commonly occurring in autoinflammatory diseases and are rarely associated with BD. Here, we present the case of a 16-year-old male with BD who presented with prolonged fever and constitutional symptoms and was found to have an aseptic splenic abscess. Rapid resolution of the symptoms along with radiological evidence of abscess shrinkage was achieved with corticosteroid therapy.

## Introduction

Behçet’s disease (BD) is a multisystem disease of unknown etiology that was initially described in 1937 as a triad of recurrent aphthosis, genital ulceration, and associated ocular disease [1]. It is more common along the ancient Silk Road with the highest reported prevalence in Turkey [2]. The manifestations are believed to be due to systemic vasculitis of nature including genetic and environmental factors, especially infectious agents [3]. An aseptic abscess (AA) is an autoinflammatory disorder characterized by necrotic lymph nodes and an abscess involving internal organs, most commonly the spleen associated with negative cultures and serologic tests, failure of antibiotic therapy, and improvement on corticosteroids [4,5]. Aseptic splenic abscesses associated with BD are rarely described in the literature [6,7].

## Case presentation

A 16-year-old boy presented with a documented intermittent fever for seven days. He had been unwell for the past few weeks, experiencing reduced appetite with nausea and vomiting. His past medical history included well-controlled bronchial asthma; otherwise, the rest of his history was unremarkable. There was no recent travel history; family history of malignancy or autoimmune diseases; contact history of tuberculosis; history of chronic cough, loss of weight, high-risk sexual behavior, and features of autoimmune disease; or history of rheumatic heart disease to suggest an autoimmune process, malignancy, or infectious process. He was discharged from the ward with the spontaneous resolution of fever in three days.

A month later, he again presented with a fever for nine days. Contrast-enhanced computerized tomography (CECT) revealed a splenic abscess. Despite the lack of serological evidence for melioidosis, he was given a trial of meropenem, which resulted in the resolution of fever for the next three months. However, the patient developed a high fever with chills and rigors again with prominent abdominal pain associated with loss of appetite. On examination, he had severe left upper quadrant tenderness with splenomegaly. In addition to the previous history, he was found to have painful oral aphthous ulcers which had developed recently. CECT at this point revealed a splenic cyst measuring 53 × 46 × 77 mm without evidence of abscess formation. His investigations including bone marrow biopsy and culture, antinuclear antibodies, Mantoux test, malaria parasite, direct antigen test, blood picture, melioidosis antibodies, retroviral studies, viral hepatitis antibodies, and two-dimensional echocardiogram were negative for a possible cause of prolonged fever. The symptoms responded to doxycycline which was started with the suspicion of typhus which is common in Sri Lanka. The patient was discharged after a week of antibiotic course despite the radiological absence of significant interval changes in the splenic abscess.

**Figure 1:**
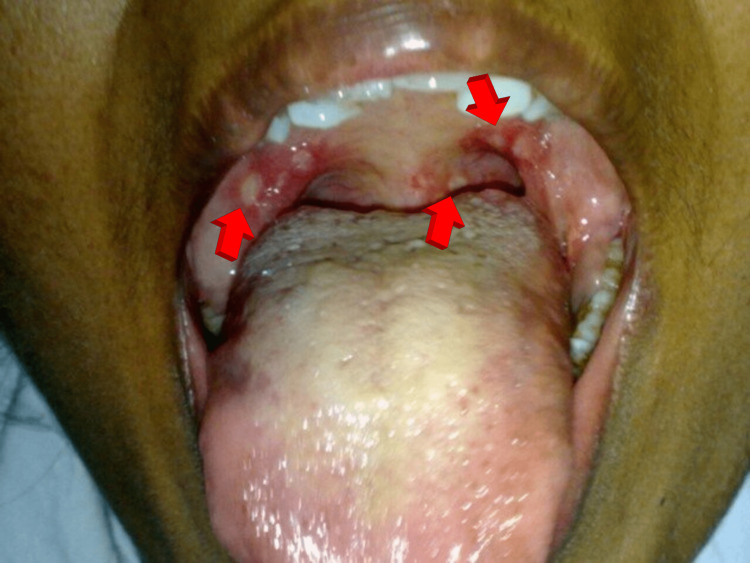
Oral aphthous ulcers.

**Figure 2:**
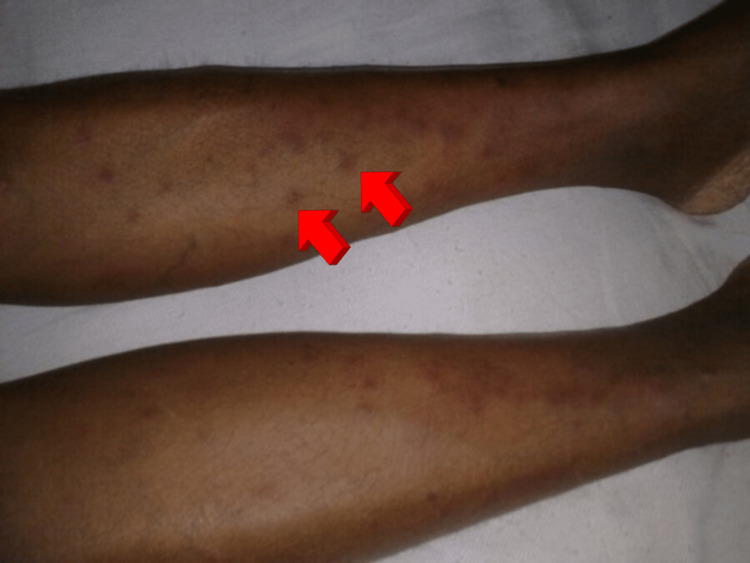
Erythema nodosum.

The patient was well for the next two months before presenting with deep vein thrombosis of the lower limbs and inferior vena cava. Thrombophilia screening including JAK2, lupus anticoagulant, anticardiolipin antibody, CD55, and CD59 on flow cytometry was negative. During this period, the patient developed oral ulcers, genital ulcers, erythema nodosum, and skin pustules (Figures 1, 2). Skin biopsy showed leukoclastic vasculitis. He denied having visual disturbances suggestive of ocular involvement.

The dermatologist diagnosed him with BD based on the diagnostic criteria of the International Study Group (ISG) for BD and was commenced on corticosteroid therapy (oral prednisolone 0.75 mg/kg/day) and anticoagulation with warfarin after a multidisciplinary meeting involving a rheumatologist, hematologist, and dermatologist. The patient was also started on calcium supplements and omeprazole. Colchicine was given for oral and genital ulcers along with a local antibiotic application. The patient showed an excellent response with the resolution of symptoms within five days of treatment initiation and remained asymptomatic during the one-year follow-up.

## Discussion

BD is an extremely rare disease in Sri Lanka and reported only in a few cases to our knowledge [8,9]. Our patient’s diagnosis was made according to the ISG diagnostic criteria based on the clinical picture and exclusion of the other diseases, as there is no single pathognomonic laboratory test to diagnose BD. The ISG criteria of BD require idiopathic recurrent aphthous ulcerations (three episodes in any 12 months) with any two of the following: recurrent genital ulceration (leaving scarring), eye lesions (anterior or posterior uveitis, presence of cells in vitreous detected by slit-lamp examination, retinal vasculitis), skin lesions (erythema nodosum, pseudofolliculitis, or papulopustular lesions, acneiform nodules in patients after adolescence and who are not using glucocorticoids), or positive pathergy test results with reading performed in 24-48 hours after the test [10,11]. HLA-B 51 is an important genetic factor in BD; however, this is neither necessary nor sufficient to diagnose the disease [12]. Our patient fulfilled the main criterion of recurrent aphthous ulcers along with genital ulcers and dermatological lesions of erythema nodosum and papulopustular lesions. However, the pathergy test was negative and eye lesions were absent.

Aseptic splenic abscesses are rarely reported in adults and present with a triad of weight loss, left upper quadrant pain, and fever in most cases [13]. Diagnosis of AA is of exclusion based on the following criteria suggested by Andre et al.: (1) deep abscess, upon radiological examination, with neutrophilic features proven by surgical pathology or aspiration when performed; (2) negative cultures and serological tests; (3) failure of the antibiotic therapy; and (4) rapid improvement with corticosteroids [14]. AA has been reported in inflammatory bowel disease, especially in Crohn’s disease, Sweet’s syndrome, and pyoderma gangrenosum, and those were ruled out by the absence of other dermatological features suggestive of the diseases [5]. This patient fulfilled the criteria with radiological evidence of splenic abscess in CECT along with negative microbiological tests and responded briefly to antibiotics only to present with recurring symptoms while demonstrating rapid response with the initiation of corticosteroids which is reported in AA associated with BD.

Management of aseptic splenic abscesses can be either pharmacological with immunosuppression or surgical with splenectomy. The literature strongly suggests the use of glucocorticoids in the treatment which results in rapid clinical resolution of symptoms followed by a slower radiological improvement [6,13,15]. There is no globally accepted therapeutic regime for the treatment of aseptic splenic abscess in BD; however, corticosteroids in the form of oral prednisolone with or without steroid-sparing agents is generally commenced as the initial therapy. If there is clinical progression despite steroid therapy, treatment can be escalated to intensified immunosuppressive therapy with cyclosporine, cyclophosphamide, or infliximab. Symptomatic management comprising pain relief and antipyretics is essential to optimize the quality of life which is otherwise affected by the multisystem involvement along with the symptoms of AA of the spleen. Colchicine is used to support the healing of oral and genital ulcers and the local application of antibiotics can be used in the setting of secondary infection. Nonsteroidal anti-inflammatory drugs are utilized for pain control. Anticoagulation is generally commenced in the presence of thrombosis [16-18]. Evidence suggests immunosuppression alone might be enough for the treatment of venous thrombosis [19,20]. However, in our patient, anticoagulation with warfarin was commenced due to venous thrombosis in multiple sites, as suggested by the hematologist. Splenectomy as a treatment option for this condition carries risks such as overwhelming postoperative infections, thrombosis, and pulmonary hypertension. The development of liver abscesses after splenectomy in BD has been described in the literature [6]. Therefore, splenectomy is not widely accepted as the initial treatment modality among medical practitioners for AA of the spleen in BD and is reserved for cases not responding to immunosuppression. Our patient demonstrated an excellent clinical response to prednisolone which did not necessitate further escalation or splenectomy. Warfarin was commenced and titrated to achieve a target of an international normalized ratio of 2-3. During the follow-up, no further thrombotic episodes occurred.

## Conclusions

Aseptic splenic abscesses are an uncommon entity and BD should be borne in mind even in the absence of the classical features as it can precede the other symptoms. When diagnosed, immunosuppression is the cornerstone of medical management and splenectomy may have a role in refractory cases. BD should not be missed in the evaluation for AA of the spleen and can be successfully managed medically with immunosuppression to avoid unnecessary splenectomies.
